# Development and validation of radiomics machine learning model based on contrast-enhanced computed tomography to predict axillary lymph node metastasis in breast cancer

**DOI:** 10.17305/bjbms.2022.7853

**Published:** 2023-03-16

**Authors:** Jieqiu Zhang, Gaofei Cao, Haowen Pang, Jin Li, Xiaopeng Yao

**Affiliations:** 1 School of Public Health, Southwest Medical University, Luzhou, China; 2 School of Medical Information and Engineering, Southwest Medical University, Luzhou, China; 3 Department of Oncology, The Affiliated Hospital of Southwest Medical University, Luzhou, China; 4 Central Nervous System Drug Key Laboratory of Sichuan Province, Southwest Medical University, Luzhou, China

**Keywords:** Breast cancer, axillary lymph node metastasis (ALNM), radiomics, machine learning, contrast-enhanced computed tomography (CECT), nomogram

## Abstract

Preoperative identification of axillary lymph node metastasis can play an important role in treatment selection strategy and prognosis evaluation. This study aimed to establish a clinical nomogram based on lymph node images to predict lymph node metastasis in breast cancer patients. A total of 193 patients with non-specific invasive breast cancer were divided into training (*n* ═ 135) and validation set (*n* ═ 58). Radiomics features were extracted from lymph node images instead of tumor region, and the least absolute shrinkage and selection operator logistic algorithm was used to select the extracted features and generate radiomics score. Then, the important clinical factors and radiomics score were integrated into a nomogram. A receiver operating characteristic curve was used to evaluate the nomogram, and the clinical benefit of using the nomogram was evaluated by decision curve analysis. We found that clinical N stage and radiomics score were independent clinical predictors. Besides, the nomogram accurately predicted axillary lymph node metastasis, yielding an area under the receiver operating characteristic curve of 0.95 (95% confidence interval 0.93–0.98) in the validation set, indicating satisfactory calibration. Decision curve analysis confirmed that the nomogram had higher clinical utility than clinical N stage or radiomics score alone. Overall, the nomogram based on radiomics features and clinical factors can help radiologists to predict axillary lymph node metastasis preoperatively and provide valuable information for individual treatment.

## Introduction

Breast cancer is the most common type of malignant tumor in women, and the average age of patients with breast cancer is decreasing [1–3]. Whether breast cancer is accompanied by axillary lymph node metastasis (ALNM) affects the subsequent treatment plans and is also a crucial prognostic factor [4, 5]. Therefore, accurate assessment of ALNM has guiding significance in the diagnosis and treatment of breast cancer.

Current methods for assessing ALNM can be either invasive or non-invasive. Due to the limitations of subjective factors such as the experience and knowledge level of clinicians, routine imaging and preoperative clinical factors result in high false-negative rate [6, 7]. Sentinel lymph node biopsy (SLNB) is the gold standard for clinical evaluation of ALNM [8], but it is invasive and can cause numerous complications that affect patients’ quality of life [9–11].

The development of artificial intelligence in the medical field has provided new possibilities for the accurate prediction of lymph node metastasis in breast cancer. Radiomics extracts the characteristic information of relevant lesions and improves the accuracy of preoperative ALNM prediction. Moreover, it is a non-invasive and low-cost method [12], and radiomics models have been widely used to predict ALNM [13–15]. However, existing studies have focused on using tumors as regions of interest (ROI) rather than lymph nodes to predict the presence of ALNM.

In this study, we developed and validated a nomogram based on ALN images for the simple and effective prediction of ALNM in breast cancer.

## Materials and methods

### Patients

Inclusion criteria were: (1) contrast-enhanced computed tomography (CECT) images of the patient’s venous phase were available; (2) patients underwent ALN dissection or SLNB; (3) available clinical data. Exclusion criteria were: (1) ALN diameter < 0.5 cm, which results in difficulties in delineating ROI in CECT images; (2) images of lymph nodes were ambiguous; (3) preoperative therapy (resection biopsy, neoadjuvant radiotherapy, or chemotherapy).

This study included 193 consecutive patients who had non-specific invasive breast cancer between February 2021 and February 2022. All patients were randomly divided into a training set (*n* ═ 135) and a validation set (*n* ═ 58), with a ratio of approximately 7:3. We obtained the patients’ baseline clinicopathological data from the medical records, and the radiologist made a comprehensive assessment based on the physical examination and CECT images, and the assessment results were cN0 (non-ALNM) and cN+ (ALNM). cN0 was defined as no regional lymph node metastases detected on CECT, whereas cN+ was defined as metastases to mobile ipsilateral level II ALNs, which depended on the radiologist’s clinical experience. The primary outcome of this study was the pathological node stage (pNx), which was determined by the results of SLNB or ALN dissection procedures.

### Contrast-enhanced CT image acquisition

All patients received preoperative contrast-enhanced chest CT examination (Philips Medical Systems, the Netherlands). The scanning method was as follows: a contrast agent (Iohexol, 320 mg/mL) was injected into the patient’s median cubital vein using a two-barrel high-pressure syringe, at a dosage of 1.0 mL/kg and a flow rate of 3.0 mL/s. The CT value of blood vessels at the level of the aortic arch was monitored after injection of the contrast agent. Enhanced CT scans were automatically triggered when the CT value reached approximately 250 HU, whereas venous phase scans were performed after a delay of 30 s. The scanning range extended from the level of the lower neck to the bottom of the thorax in the supine position.

**Table 1:** Clinical characteristics of patients

**Characteristics**	**Total (*n* ═ 193)**		***p***
	**pN0 (*n* ═ 97)**	**pN+ (*n* ═ 96)**	
Age (y)	51.8 ± 8.7	49.4 ± 9.9	0.075
Tumor grade			<0.001
I	28 (28.9)	1 (1.0)	
II	62 (63.9)	57 (59.4)	
III	7 (7.2)	38 (39.6)	
Histopathologic subtype			0.721
Invasive ductal carcinoma	92 (94.9)	93 (96.9)	
Invasive lobular carcinoma	5 (5.2)	3 (3.1)	
Clinical T stage (%)			<0.001
T1	32 (34.3)	13 (14.4)	
T2	60 (60.6)	57 (58.8)	
T3	5 (5.0)	26 (26.8)	
Clinical N stage (%)			<0.001
N0	72 (74.2)	5 (5.2)	
N+	25 (25.8)	91 (94.8)	
ER status (%)			0.455
Negative	31 (32.0)	25 (26.0)	
Positive	66 (68.0)	71 (74.0)	
PR status (%)			0.134
Negative	36 (37.1)	25 (26.0)	
Positive	61 (62.9)	71 (74.0)	
HER2 status (%)			0.812
Negative	58 (59.8)	60 (62.5)	
Positive	39 (40.2)	36 (37.5)	
Ki-67 status (%)			0.249
<30%	49 (50.5)	40 (41.7)	
≥30%	48 (49.5)	56 (58.3)	

Data in parentheses are percentages; *p* values were derived from the univariate analysis between each of characteristic and axillary lymph node status. HER2: Human epidermal growth factor receptor 2; ER: Estrogen receptor; PR: Progesterone receptor.

### The workflow

**Figure 1.**
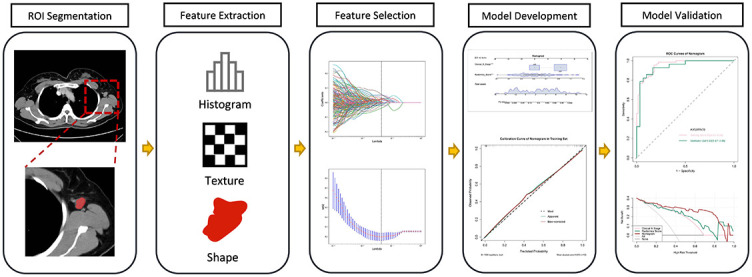
**The workflow of the radiomics analysis.** ROI: Region of interest; ROC: Receiver operating characteristics curve; MSE: Mean squared error.

The workflow of the radiomics analysis included ROI segmentation, feature extraction, feature selection, model development, and model validation (Figure 1).

### Segmentation and features extraction

**Figure 2.**
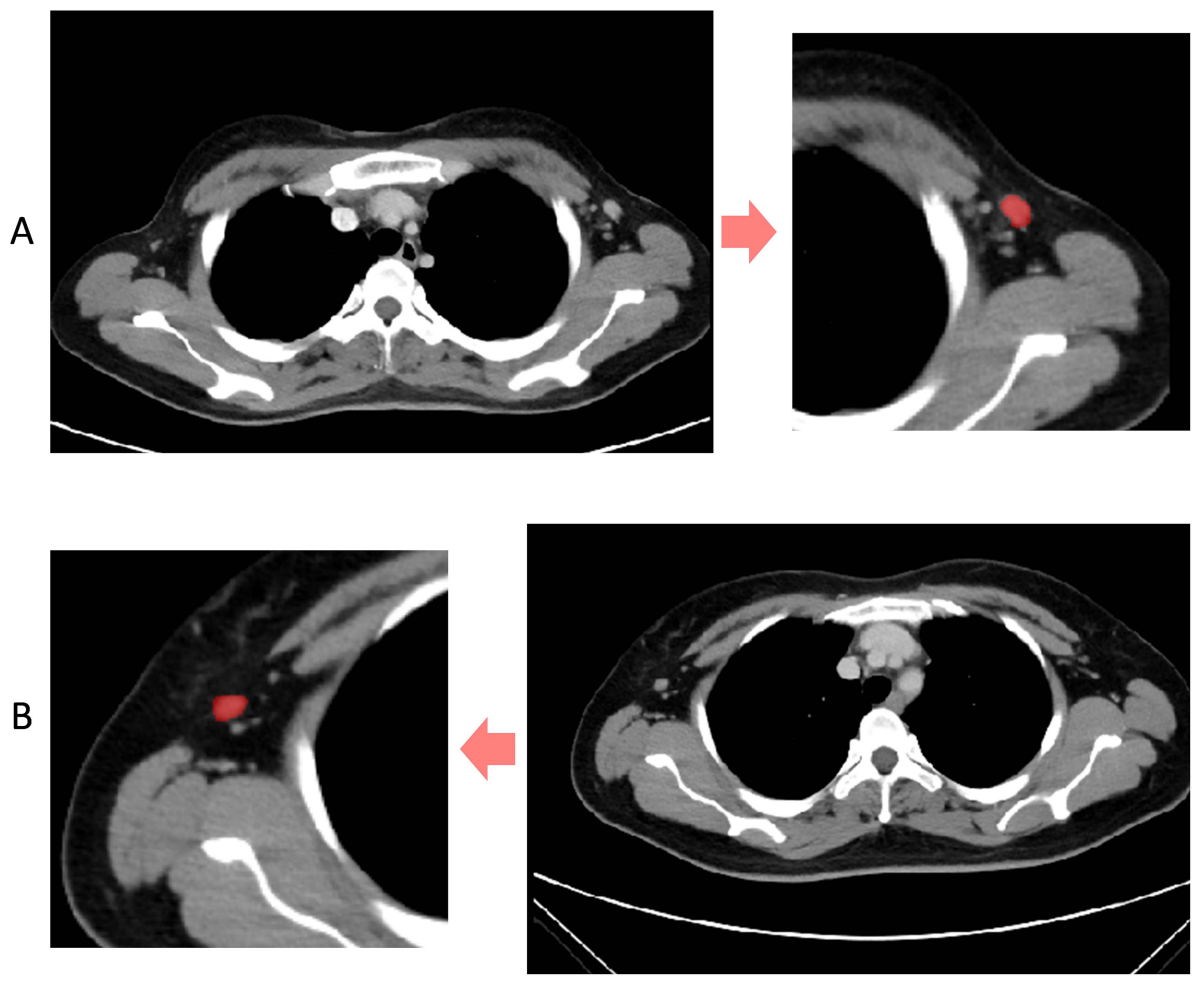
**Segmentation of the region of interest.** (A) Shows the segmentation of a non-metastatic axillary lymph node; (B) Shows the segmentation of a metastatic axillary lymph node.

Contrast-enhanced 2D images of the venous phase were obtained from Digital Imaging and Communications in Medicine (DICOM). We selected images with a slice thickness of 5 mm and the ROI segmentation was performed on the original CECT images using ITK-SNAP software (http://www.itksnap.org) (Figure 2) [16]. All manual segmentation of the CECT images was performed by two practicing experienced radiologists (Y.W., with 11 years of CECT imaging experience; Y.T., with 2 years of CECT imaging experience) who were blinded to the patients’ clinical information. For the radiomics feature extraction of the ROI images, we used Pyradiomics, an open-source package based on Python.

### Radiomics score construction and performance assessment

We normalized all variables and measured correlations between the features using Pearson algorithm. If the correlation coefficient between the two features was greater than 0.9, one of the features was excluded. The least absolute shrinkage and selection operator (LASSO) algorithm was applied to select the extracted features. The selected features were weighted by their respective LASSO coefficients and the linear combination was used to calculate the radiomics score. The radiomics score performance was assessed in both the training and validation set and the area under the curve (AUC) of the receiver operator characteristic (ROC) curve was used to assess the discriminative ability of the radiomics score.

### Nomogram development and validation

The radiomics score and other clinical predictors were tested using a multivariate logistic regression algorithm in the training set. We then selected independent predictors for developing the nomogram. Calibration was assessed using a calibration curve along with the Hosmer–Lemeshow test to evaluate the goodness-of-fit of the nomogram. Additionally, the ROC curve and decision curve analysis were implemented to determine discriminative ability and clinical utility.

### Ethical statement

The study was conducted in accordance with the Declaration of Helsinki, and approved by the Ethics Committee of the Affiliated Hospital of Southwest Medical University (protocol code KY2022216, 20 June 2022). The requirement for written informed consent was waived.

### Statistical analysis

Normally distributed data were analyzed using an independent t-test, while categorical variables were evaluated using the chi-squared test. Except for the LASSO algorithm, which was performed using Python software, all statistical analyses were conducted using R statistical software, version 4.2.0. All statistical tests were two-tailed, and *p* < 0.05 was deemed statistically significant.

## Results

### Patient clinical characteristics

The results of clinical and histopathological characteristics of patients with pN0 (*n* ═ 97) and pN+ (*n* ═ 96) are presented in Table 1. Univariate analysis revealed that when pathological outcome was the outcome variable, only clinical T stage, clinical N stage, and tumor stage showed statistical significance.

**Figure 3.**
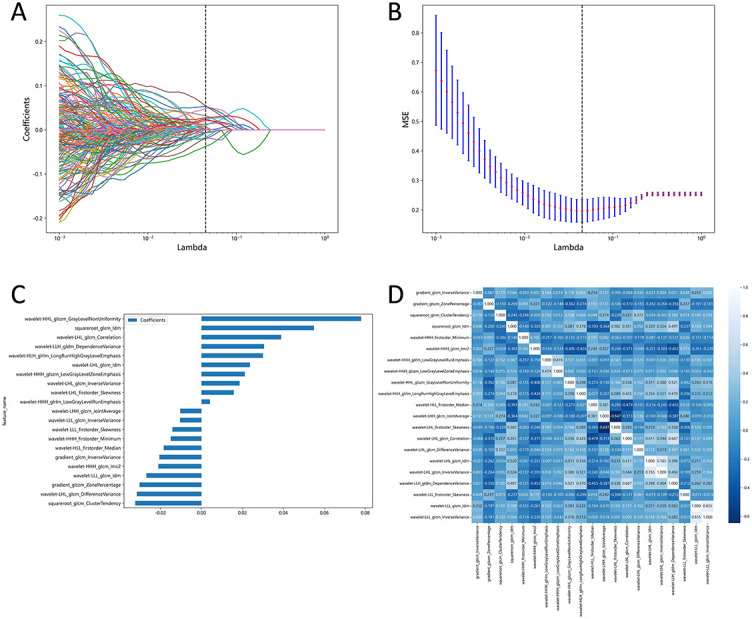
(A) The LASSO coefficient profiles of radiomics features; (B) The feature selection by the LASSO model with tuning parameter (λ) using 5-fold cross-validation via minimum criteria. The *x*-axis shows lambda, and the *y*-axis shows the mean squared error (MSE); (C) Selected optimal features and LASSO coefficients; (D) Heatmap shows the correlation coefficient between the radiomics features selected by the LASSO.

### Features selection

We customized the mode of features extraction based on Pyradiomics and set preferences for image type, feature class, and setting. In total, 1,218 features were extracted from each CECT image, including 96 original features and 1,122 wavelet features. Original features included FirstOrder (*n* ═ 18), shape-based (2D; *n* ═ 10), gray level dependence matrix (*n* ═ 14), gray level run length matrix (*n* ═ 16), gray level cooccurrence matrix (*n* ═ 22), and gray level size zone matrix (*n* ═ 16). Wavelet features were based on the original features and were derived through various filters (Wavelet, LoG, etc.). Besides, the Pearson correlation coefficient analysis was performed on the wavelet features. Ultimately, 387 radiomics features were obtained for each patient. LASSO logistic regression was used to further select the optimal features, and we finally obtained 21 potential radiomics predictors. The details of the LASSO features selection are displayed in Figure 3.

### Radiomics signature construction and performance assessment

**Figure 4.**
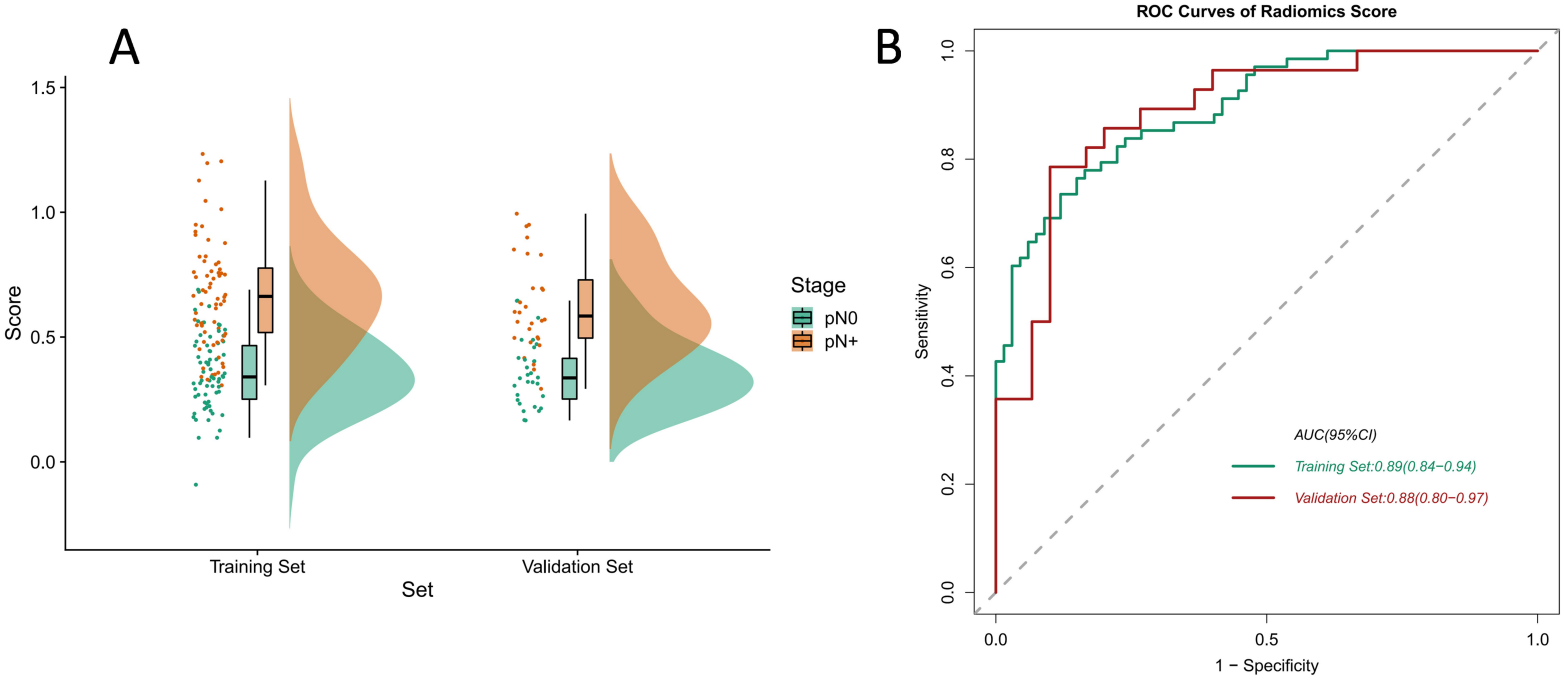
(A) The raincloud plot visualizes the score of radiomics signature. It shows the sample distribution locations and interval sample densities for the training and validation sets of radiomics features; (B) ROC curves of radiomics score for the training and validation sets. ROC: Receiver operating characteristics curve; AUC: Area under the receiver operating characteristic curve.

The formula for the radiomics score was developed according to the feature coefficients, and the calculations are shown in Table S1. We discovered that when patients had different pathological outcomes, the radiomics score was statistically different in the training and validation set, thereby confirming the discriminative ability of the radiomics signature. We used the Raincloud plot to visualize the different distributions of the samples (Figure 4A) and plotted the ROC curve to express the performance of the radiomics score. The AUC value in the validation set reached 0.88 (95% CI 0.80–0.97; Figure 4B).

### Nomogram development and validation

**Figure 5.**
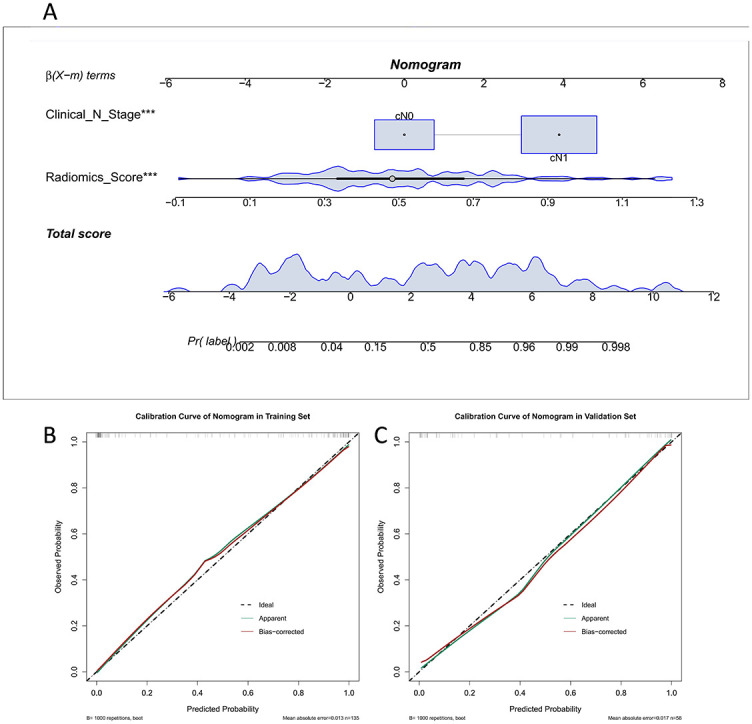
(A) Nomogram based on radiomics score and clinical N stage,*** represents *p* < 0.001; (B) Calibration curves of nomogram, the *x*-axis represents the predicted probability of ALNM estimated by the nomogram, and the *y*-axis represents the actual ALNM probability. ALNM: Axillary lymph node metastasis.

Univariate analysis was used to select statistically significant clinicopathological features (*p* < 0.05). Combined with the radiomics score for the multivariate logistic regression analysis, the results indicated that the clinical N stage and radiomics score were statistically significant (*p* < 0.001), as the nomogram consisted of the above factors. A goodness-of-fit test was performed on the nomogram and a calibration curve was drawn to show that the predicted probability of the nomogram for ALNM corresponded closely with actual observations (*p* > 0.05; Figure 5B and 5C).

**Figure 6.**
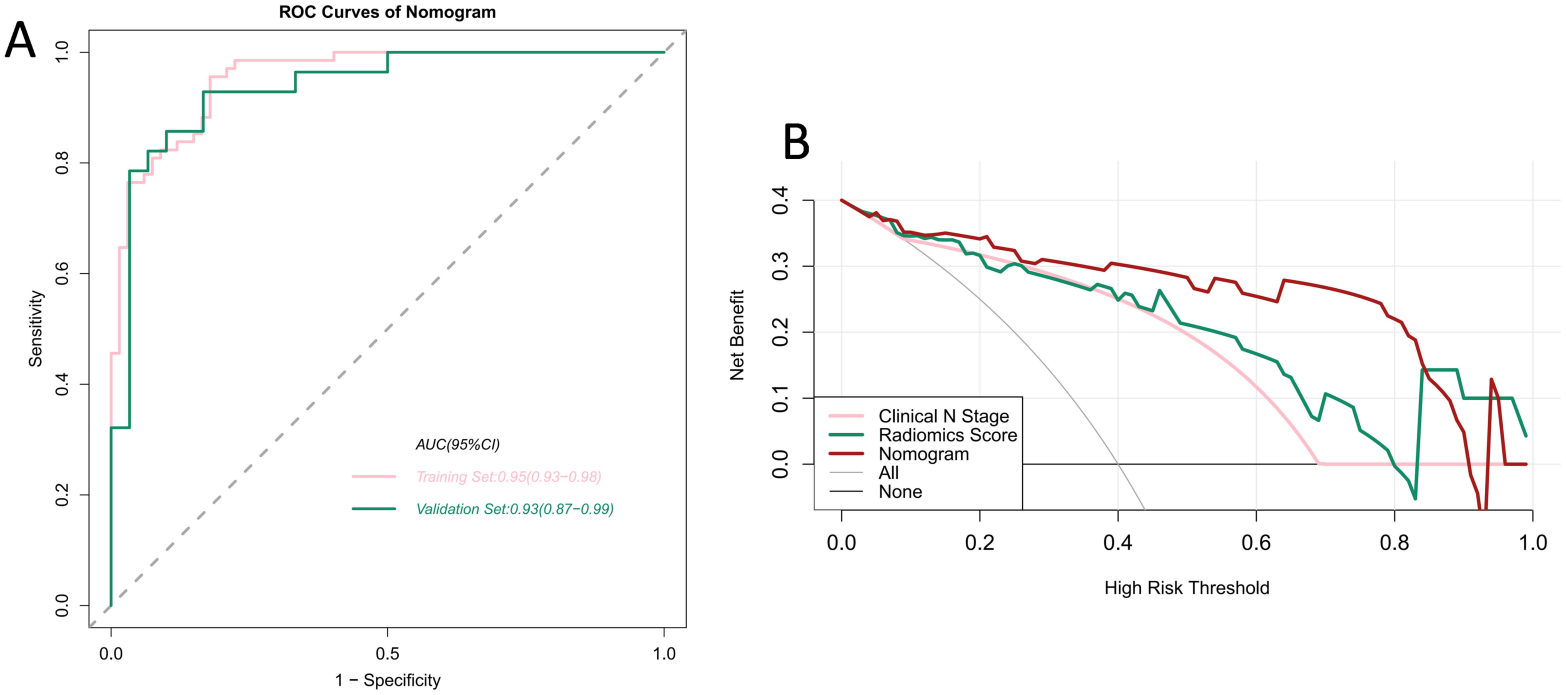
(A) ROC curves of nomogram for the training and validation sets; (B) DCA of nomogram in the validation set. The *x*-axis represents the threshold probability, the *y*-axis represents net benefit. The gray and black lines represent the hypothesis that all breast cancer patients are pN+ and pN0, respectively. When the high-risk threshold is less than 0.9, the net benefit of the nomogram is higher than the radiomics score and clinical N stage. ROC: Receiver operating characteristics curve; AUC: Area under the receiver operating characteristic curve; DCA: Decision curve analysis.

We verified the predictive effect of the nomogram, which reached peak AUC values of 0.95 (95% CI 0.93–0.98) and 0.93 (95% CI 0.87–0.99) in the training and validation sets, respectively. This demonstrates the outstanding predictive ability of the nomogram. The detailed statistical results for discriminating pN0 and pN+ patients are summarized in Table 2, and the corresponding ROC curves are shown in Figure 6A. We plotted the decision curves of the models in the validation set and found that the nomogram had superior clinical benefits compared to the radiomics score (Figure 6B).

**Table 2:** Performance of models for predicting axillary lymph node metastasis in breast cancer patients

**Training set**	**AUC (95% CI)**	**SEN (%)**	**SPE (%)**	**ACC (%)**	**PPV (%)**	**NPV (%)**
Radiomics score	0.89 (0.84–0.94)	0.74	0.88	0.81	0.86	0.77
Nomogram	0.95 (0.93–0.98)	0.96	0.82	0.89	0.84	0.95
**Validation set**	**AUC (95% CI)**	**SEN (%)**	**SPE (%)**	**ACC (%)**	**PPV (%)**	**NPV (%)**
Radiomics score	0.88 (0.80–0.97)	0.78	0.90	0.84	0.88	0.82
Nomogram	0.93 (0.87–0.99)	0.93	0.83	0.88	0.84	0.93

AUC: Area under the receiver operating characteristic curve; SEN: Sensitivity; SPE: Specificity; ACC: Accuracy; PPV: Positive predictive value; NPV: Negative predictive value.

## Discussion

The ALNM of breast cancer is a crucial aspect that affects the prognosis of patients. It is also a key indicator for determining the stage of breast cancer and guiding neoadjuvant chemotherapy. In this study, we constructed a radiomics score based on lymph node radiomics features and then added clinicopathological features to generate a clinical nomogram. In the validation set, the nomogram was constructed by adding a combination of clinicopathological factors, and the AUC reached 0.95. This demonstrates that clinical risk factors in ALNM can compensate for the insufficient predictive power of radiomics features alone. Decision curve analysis showed that the clinical utility of the nomogram was higher than that of the simple clinical N stage and the radiomics score. Therefore, this model can be used to accurately predict the ALN status and enhance clinical decision making for treatment strategies, and thus has great potential for clinical application.

In clinical practice, the preoperative diagnosis of ALNM is based not only on tumor size but also on various clinical features in lymph node images, including long-axis diameter, short-axis diameter, cortical thickness, ALN fatty hilum, and ALN shape. Several studies have validated the diagnostic performance of the above individual features. For example, cortical thickness (> 3 mm) and non-fatty hilum of multidetector CT were independent predictors of ALNM [17]. However, that particular study was based on a small sample, and it remains to be verified whether its results can be universally applied. One study reported that lymph node size was associated with ALNM in breast cancer [18], but in another it was not an independent predictor of ALNM [19]. Regarding the shape of lymph nodes, although bean-shaped lymph nodes have been reported to exhibit significant metastases, one study found that clear-type ALNs may also be predictors of metastasis, while “crescent” and annular nodes are non-metastatic [19]. The above findings suggest that various clinical features of lymph node images pose uncertainties in predicting malignant metastasis. It is difficult to determine from images alone whether ALNs have metastasized, and accuracy generally depends on the clinician’s experience. Therefore, there is a need for a universal and accurate method to predict ALNM in cases of breast cancer.

To date, several academics have made preoperative predictions for ALNM based on the radiomics of magnetic resonance imaging, positron emission tomography–computed tomography, and ultrasonography [3, 15, 20]. The combined models have achieved good results, but most of them extracted the quantitative features of the lesion area to predict ALNM and did not consider the lymph node images. We found that models based on the radiomic features of lesions were generally less powerful, and their predictive power increased sharply when additional clinical risk factors were added. Therefore, some scholars believe that the predictive power of radiomic features may be limited. Existing studies have used lymph node images for features extraction and produced combined models that incorporate the features of lesion images [21, 22] and have strong predictive ability. However, the difference in predictive ability between radiomics models and combined models is small, confirming the predictive ability of radiomics features.

In our study, radiomics features of lymph node images were extracted and combined with independent clinical predictors for preoperative prediction of ALNM. The prediction results were similar as those in Yang’s study (AUC ═ 0.94), indicating that the radiomics features of lymph node images are highly effective. Thus, ALNM can be comprehensively predicted by combining the radiomics features of lesions and lymph node images.

There are some limitations to our study. First, it is a retrospective study and the sample data are from a single medical institution. Thus, the generalizability and robustness of the model are poor. To address this issue, large sample studies with different groups of patients from multiple medical institutions are required. Besides, we only preliminarily explored the feasibility of using the radiomics features of lymph node images to predict ALNM. The sample size was relatively small and prone to underfitting, and a larger sample size is required for machine learning training. Third, interobserver agreement was not assessed in our study due to a lack of evaluation of interrater, test-retest, and intrarater reliability; however, a subsequent study will incorporate it into the study.

## Conclusion

Nomogram is helpful for individual prediction of patients with breast cancer and could assist clinicians to perform preoperative assessments and make better clinical decisions. Future research will encompass more breast cancer patient databases, integrate more clinical factors, and further improve the predictive model.

## Supplemental Data

**Table S1:** Radiomics score formula

**Model**	**Formula**
Radiomics score	Radiomics score ═ 0.5057222023348106
	−0.020572 * gradient_glcm_InverseVariance
	−0.030204 * gradient_glszm_ZonePercentage
	−0.032331 * squareroot_glcm_ClusterTendency
	+0.054891 * squareroot_glcm_Idm
	−0.014994 * wavelet-HHH_firstorder_Minimum
	−0.021038 * wavelet-HHH_glcm_Imc2
	+0.004121 * wavelet-HHH_glrlm_LowGrayLevelRunEmphasis
	+0.021182 * wavelet-HHH_glszm_LowGrayLevelZoneEmphasis
	+0.077968 * wavelet-HHL_glszm_GrayLevelNonUniformity
	+0.030022 * wavelet-HLH_glrlm_LongRunHighGrayLevelEmphasis
	−0.018456 * wavelet-HLL_firstorder_Median
	−0.010342 * wavelet-LHH_glcm_JointAverage
	+0.015771 * wavelet-LHL_firstorder_Skewness
	+0.038912 * wavelet-LHL_glcm_Correlation
	−0.031525 * wavelet-LHL_glcm_DifferenceVariance
	+0.023642 * wavelet-LHL_glcm_Idm
	+0.018603 * wavelet-LHL_glcm_InverseVariance
	+0.030586 * wavelet-LLH_gldm_DependenceVariance
	−0.014144 * wavelet-LLL_firstorder_Skewness
	−0.026837 * wavelet-LLL_glcm_Idm
	−0.010474 * wavelet-LLL_glcm_InverseVariance

**Table S2:** Difference examination between training and validation sets

**Characteristics**	**Training set**	**Validation set**	***p***
	**(*n* ═ 135)**	**(*n* ═ 58)**	
Age (y)	50.20 ± 9.32	51.50 ± 9.57	0.377
Tumor Grade			0.156
I	19 (14.1)	10 (17.2)	
II	89 (65.9)	30 (51.7)	
III	27 (20.0)	18 (31.0)	
Histopathologic subtype			0.209
Invasive ductal carcinoma	131 (97.0)	54 (93.1)	
Invasive lobular carcinoma	4 (3.0)	4 (6.9)	
Clinical T stage			0.498
T1	31 (0.23)	14 (0.23)	
T2	79 (0.59)	38 (0.64)	
T3	24 (0.18)	7 (0.13)	
Clinical N stage			0.421
N0	56 (0.42)	21(0.36)	
N+	78 (0.58)	38(0.64)	
ER status			0.763
Negative	38 (0.28)	18 (0.31)	
Positive	96 (0.72)	41 (0.69)	
PR status			0.829
Negative	43 (0.32)	18 (0.30)	
Positive	91 (0.68)	41 (0.70)	
HER2 status			0.768
Negative	81 (0.60)	37 (0.63)	
Positive	53 (0.40)	22 (0.37)	
Ki-67			0.161
<30%	47 (0.35)	27 (0.46)	
≥30%	87 (0.65)	32 (0.54)	
Pathological N stage			0.41
N0	70 (0.52)	27 (0.46)	
N+	64 (0.48)	32 (0.54)	

HER2: Human epidermal growth factor receptor 2; ER: Estrogen receptor; PR: Progesterone receptor.

## References

[1] Smolarz B, Nowak AZ, Romanowicz H (2022). Breast cancer—epidemiology, classification, pathogenesis and treatment (review of literature). Cancers (Basel).

[2] Siegel RL, Miller KD, Fuchs HE, Jemal A (2022). Cancer statistics, 2022. CA Cancer J Clin.

[3] DeSantis CE, Ma J, Gaudet MM, Newman LA, Miller KD, Goding Sauer A (2019). Breast cancer statistics, 2019. CA Cancer J Clin.

[4] Fillon M (2022). Breast cancer recurrence risk can remain for 10 to 32 years. CA Cancer J Clin.

[5] Giuliano AE, Connolly JL, Edge SB, Mittendorf EA, Rugo HS, Solin LJ (2017). Breast cancer—major changes in the American Joint Committee on Cancer eighth edition cancer staging manual. CA Cancer J Clin.

[6] Zhang X, Liu Y, Luo H, Zhang J (2020). PET/CT and MRI for identifying axillary lymph node metastases in breast cancer patients: systematic review and meta-analysis. J Magn Reson Imaging.

[7] Samiei S, de Mooij CM, Lobbes MBI, Keymeulen K, van Nijnatten TJA, Smidt ML (2021). Diagnostic performance of noninvasive imaging for assessment of axillary response after neoadjuvant systemic therapy in clinically node-positive breast cancer: a systematic review and meta-analysis. Ann Surg.

[8] Coutant C, Olivier C, Lambaudie E, Fondrinier E, Marchal F, Guillemin F (2009). Comparison of models to predict nonsentinel lymph node status in breast cancer patients with metastatic sentinel lymph nodes: a prospective multicenter study. J Clin Oncol.

[9] Ashikaga T, Krag DN, Land SR, Julian TB, Anderson SJ, Brown AM (2010). Morbidity results from the NSABP B-32 trial comparing sentinel lymph node dissection versus axillary dissection. J Surg Oncol.

[10] DiSipio T, Rye S, Newman B, Hayes S (2013). Incidence of unilateral arm lymphoedema after breast cancer: a systematic review and meta-analysis. Lancet Oncol.

[11] Liu CQ, Guo Y, Shi JY, Sheng Y (2009). Late morbidity associated with a tumour-negative sentinel lymph node biopsy in primary breast cancer patients: a systematic review. Eur J Cancer.

[12] Mayerhoefer ME, Materka A, Langs G, Häggström I, Szczypiński P, Gibbs P (2020). Introduction to radiomics. J Nucl Med.

[13] Zheng X, Yao Z, Huang Y, Yu Y, Wang Y, Liu Y (2020). Deep learning radiomics can predict axillary lymph node status in early-stage breast cancer. Nat Commun.

[14] Song BI (2021). A machine learning-based radiomics model for the prediction of axillary lymph-node metastasis in breast cancer. Breast Cancer.

[15] Zhu Y, Yang L, Shen H (2021). Value of the application of CE-MRI radiomics and machine learning in preoperative prediction of sentinel lymph node metastasis in breast cancer. Front Oncol.

[16] Yushkevich PA, Piven J, Hazlett HC, Smith RG, Ho S, Gee JC (2006). User-guided 3D active contour segmentation of anatomical structures: significantly improved efficiency and reliability. Neuroimage.

[17] Chen CF, Zhang YL, Cai ZL, Sun SM, Lu XF, Lin HY (2019). Predictive value of preoperative multidetector-row computed tomography for axillary lymph nodes metastasis in patients with breast cancer. Front Oncol.

[18] March DE, Wechsler RJ, Kurtz AB, Rosenberg AL, Needleman L (1991). CT-pathologic correlation of axillary lymph nodes in breast carcinoma. J Comput Assist Tomogr.

[19] Kutomi G, Ohmura T, Satomi F, Takamaru T, Shima H, Suzuki Y (2014). Lymph node shape in computed tomography imaging as a predictor for axillary lymph node metastasis in patients with breast cancer. Exp Ther Med.

[20] Yu Y, Tan Y, Xie C, Hu Q, Ouyang J, Chen Y (2020). Development and validation of a preoperative magnetic resonance imaging radiomics-based signature to predict axillary lymph node metastasis and disease-free survival in patients with early-stage breast cancer. JAMA Netw Open.

[21] Yu Y, He Z, Ouyang J, Tan Y, Chen Y, Gu Y (2021). Magnetic resonance imaging radiomics predicts preoperative axillary lymph node metastasis to support surgical decisions and is associated with tumor microenvironment in invasive breast cancer: a machine learning, multicenter study. EBioMedicine.

[22] Cheng J, Ren C, Liu G, Shui R, Zhang Y, Li J (2022). Development of high-resolution dedicated pet-based radiomics machine learning model to predict axillary lymph node status in early-stage breast cancer. Cancers (Basel).

[23] Yang C, Dong J, Liu Z, Guo Q, Nie Y, Huang D (2021). Prediction of metastasis in the axillary lymph nodes of patients with breast cancer: a radiomics method based on contrast-enhanced computed tomography. Front Oncol.

